# Dopamine Modulates Motor Control in a Specific Plane Related to Support

**DOI:** 10.1371/journal.pone.0155058

**Published:** 2016-05-04

**Authors:** Marc Herbin, Caroline Simonis, Lionel Revéret, Rémi Hackert, Paul-Antoine Libourel, Daniel Eugène, Jorge Diaz, Catherine de Waele, Pierre-Paul Vidal

**Affiliations:** 1 UMR 7179 MNHN/CNRS, Museum National d’Histoire Naturelle, Dpt EGB, CP 55, 57 rue Cuvier 75231 Paris cedex 05, France; 2 MENESR, DEPP, 61–65 rue Dudot 75015 Paris, France; 3 LJK, CNRS UMR 5224 INRIA/UJF, INRIA Rhône-Alpes, 655 av de l’Europe, 38330 Montbonnot, France; 4 SLEEP Physiopathologie des réseaux neuronaux du cycle sommeil, Centre de Recherche en Neurosciences de Lyon, INSERM U1028—CNRS UMR5292, Faculté de Médecine Laennec, 7 rue Guillaume Paradin, 69372 LYON Cedex 08 France; 5 Centre de Neurophysique, Physiologie, Pathologie, Université Paris Descartes-CNRS UMR-8119, 45 rue des Saint-Pères, 75270 Paris cedex 06, France; 6 Centre de Psychiatrie et Neurosciences, INSERM UMR 894—Université Paris Descartes, 2ter, rue d'Alésia, 78014 Paris, France; 7 COGNAC-G Université Paris Descartes-CNRS UMR-MD-SSA, 45 rue des Saint-Pères, 75270 Paris cedex 06, France; Scientific Institute Foundation Santa Lucia, ITALY

## Abstract

At the acute stage following unilateral labyrinthectomy (UL), rats, mice or guinea pigs exhibit a complex motor syndrome combining circling (HSCC lesion) and rolling (utricular lesion). At the chronic stage, they only display circling, because proprioceptive information related to the plane of support substitutes the missing utricular information to control posture in the frontal plane. Circling is also observed following unilateral lesion of the mesencephalic dopaminergic neurons by 6- hydroxydopamine hydrobromide (6-OHDA rats) and systemic injection of apomorphine (APO rats). The resemblance of behavior induced by unilateral vestibular and dopaminergic lesions at the chronic stage can be interpreted in two ways. One hypothesis is that the dopaminergic system exerts three-dimensional control over motricity, as the vestibular system does. If this hypothesis is correct, then a unilateral lesion of the nigro-striatal pathway should induce three-dimensional motor deficits, i.e., circling and at least some sort of barrel rolling at the acute stage of the lesion. Then, compensation could also take place very rapidly based on proprioception, which would explain the prevalence of circling. In addition, barrel rolling should reappear when the rodent is placed in water, as it occurs in UL vertebrates. Alternatively, the dopaminergic network, together with neurons processing the horizontal canal information, could control the homeostasis of posture and locomotion specifically in one and only one plane of space, *i*.*e*. the plane related to the basis of support. In that case, barrel rolling should never occur, whether at the acute or chronic stage on firm ground or in water. Moreover, circling should have the same characteristics following both types of lesions. Clearly, 6-OHDA and APO-rats never exhibited barrel rolling at the acute stage. They circled at the acute stage of the lesion and continued to do so three weeks later, including in water. In contrast, UL-rats, exhibited both circling and barrel rolling at the acute stage, and then only circled on the ground. Furthermore, barrel rolling instantaneously reappeared in water in UL rats, which was not the case in 6-OHDA and APO-rats. That is, the lesion of the dopaminergic system on one side did not compromise trim in the pitch and roll planes, even when proprioceptive information related to the basis of support was lacking as in water. Altogether, these results strongly suggest that dopamine does not exert three-dimensional control of the motor system but regulates postural control in one particular plane of space, the one related to the basis of support. In contrast, as previously shown, the vestibular system exerts three-dimensional control on posture. That is, we show here for the first time a relationship between a given neuromodulator and the spatial organization of motor control.

## Introduction

The vestibular system exerts a tridimensional control on posture and movement [1,2]: the horizontal canal information (HSCC) stabilizes the head-neck ensemble in a plane parallel to the plane of sustentation, including the detection of the straight-ahead direction. The utricular information stabilizes the orientation of the head neck ensemble in the frontal and sagittal plane including the detection of the gravitational vertical. Accordingly, when all vestibular sensors are lesioned on one side (UL rat), the postural syndrome observed, on ground at the acute stage, combines two types of deficits: a- a rotation of the head about the cervical column axis and circling (HSCC lesion); b- a lateral tilt of the head-neck ensemble about the thoracic column axis and rolling (utricular lesion). Later on, UL rats, mice or guinea pigs only display circling, presumably because proprioceptive information related to the plane of support substitutes for the missing utricular information to control posture in the frontal plane. However, as soon as UL rats, mice or guinea pig are placed in a tank of water, a violent barrel rolling reappears due to the disappearance of the proprioceptive information i.e. the three-dimensional nature of the vestibular deficit reappears (P.-P. Vidal and others, personal observation).

Interestingly, unilateral dopaminergic lesion also induces a rotation of the head about the axis of the cervical column towards the lesion side and a circling behavior [3–7]. As early as 1968, it was shown in a seminal paper by Ungerstedt [8] that unilateral degeneration of the whole nigro-neostriatal dopamine (DA) neuron system in rats, after intracerebral injection of 6-hydroxy-DA (6-OHDA) into the substantia nigra, produced marked motor asymmetries with turning toward the side ipsilateral to the lesion. Furthermore, a single unilateral injection of DA or unilateral application of DA crystals into the NCP, which is richly innervated by DA nerve terminals was found to produce an asymmetric posture and turning contralateral to the side of application by the same author [9, 10]. These papers showed for the first time that the nigro-neostriatal DA neurons were of considerable importance for posture and generalized motor function.

The resemblance between the consequences of a unilateral vestibular and dopaminergic lesion on motor behavior can be interpreted in two ways. One hypothesis is that the dopaminergic system exerts three-dimensional control over motricity, as the vestibular system does. If this hypothesis is correct, then a unilateral lesion of the nigro-striatal pathway should induce three-dimensional motor deficits, i.e., circling and at least some sort of barrel rolling at the acute stage of the lesion. Then, compensation of the postural syndrome in the frontal plane could also take place very rapidly, as in UL rats due to limbs proprioceptive information related to ground support, which would explain the prevalence of circling. In addition, barrel rolling should reappear when the rodents with unilateral dopaminergic lesions are placed in water due to the disappearance of proprioception. Alternatively, the dopaminergic network, together with neurons processing the horizontal canal information, could control the homeostasis of posture and locomotion specifically in one and only one plane of space, *i*.*e*. the plane related to the basis of support. In that case, barrel rolling should never occur, whether at the acute or chronic stage on firm ground or in water. Moreover, circling should have the same characteristics following both types of lesions. This paper is an attempt to address these questions by comparing quantitatively in three dimensions, the motor behavior of rats following unilateral vestibular and dopaminergic lesions both on the ground and in water.

## Materials and Methods

### Animals

The animals used in this study were 8-week-old male Wistar rats (Elevage Janvier, France). They were housed in groups of three per cage in a room with constant temperature (22°C), under 12:12 light- dark cycle, and with food pellets and water *ad libitum*. Data were collected from four wild type rats used as controls, eight rats with (left or right) unilateral lesions in the *Substantia Nigra pars compacta*, and five hemi-labyrinthectomized rats (left side).

### Surgeries

All surgeries were performed in deeply anesthetized rats (halothane). All studies were carried out in accordance with European Community Council directive of November 24th, 1986, and following the procedures issued by French Ministère de l’Agriculture. All efforts were made to minimize animal suffering and to reduce the number of animals used. All experiments were performed in Animal Research Facility under the approval ID B-75-1015 delivered by "Direction des Services Vétérinaires de la Préfecture de Paris" (France) and approved by a review board of Université Paris Descartes.

#### 6-OHDA lesion

**Fig 1:**
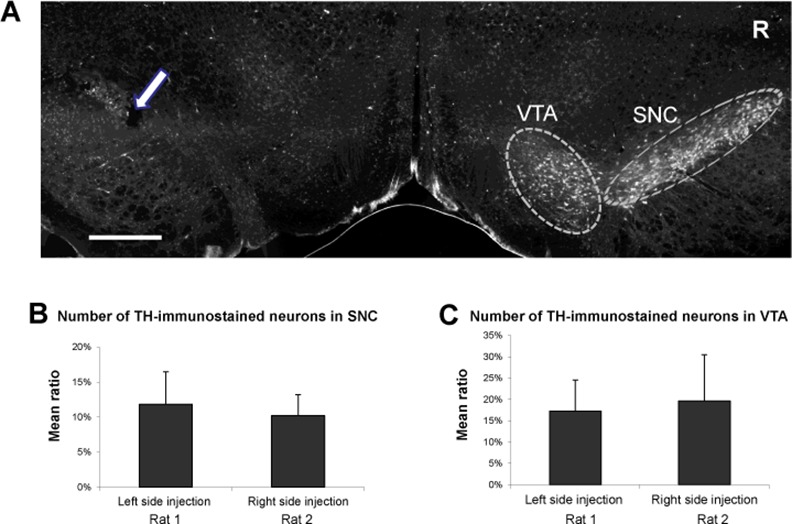
Fluorescent immunostaining of α-tyrosine hydroxylase (TH) in substantia nigra pars compacta (SNC) and ventral tegmentum area (VTA) of two rats injected with 6-OHDA. **(A)** Digital photograph illustrating typical TH immunostaining obtained in SNC and VTA of a rat injected on the left side: the place reached by the extremity of the 6-OHDA cannula is shown by an arrow. TH immunostaining is much weaker on the left side than on the right side indicated by letter R (Scale bar: 800 μm). **(B), (C)** Graphs giving the mean number (in %) of TH-immunostained neurons per SCN (B) and per VTA (C) between injected side and opposite side. The number of TH-immunostained neurons was measured on both sides for each brain coronal sections containing the SNC and/or the VTA and a ratio was calculated. Then for each nucleus, the mean percentage was calculated as the mean of ratios of immunostained neuron number. In both nuclei, neuron number is much lower on the injected side than on the other side.

Thirty minutes prior to surgery, the rats received an injection of desmethylimipramine (15 mg/kg ip) to protect noradrenergic neurons from the neurotoxic effects of 6- hydroxydopamine hydrobromide (6- OHDA; Sigma Chemical Co.). Under sodium pentobarbital (Somnotol) anesthesia (25 mg/kg), they were positioned in a Kopf stereotoxic instrument and received an infusion of 6-OHDA (8 ti% in 4 <J of 0.9% saline and 0.1% ascorbic acid) at a rate of 1 iA/mm through a 26-ga. stainless steel cannula (outer diameter, 0.045 cm). The cannula was left in place for a further 3 min to allow for passive diffusion away from the tip. Doses of 6-OHDA are expressed as the weight of the base. The cannula was aimed at the left A9 region of the *substantia nigra* (SN) for half of the rats and at the right A9 region for the other half. The coordinates were A 4.4, L 0.9, and V 7.2 for rats weighing 150–200 g and A 4.8, L 1.5, and V 7.5 for rats weighing 200–250 g [11]. The lesion sites were verified at the end of the study using serial coronal sections of the brain stained with cresyl violet. In a second time, a quantification of TH immunostained neurons was done in SN on both sides to verify that the the number of stained neurons was lower in the injected side than the other side (**Fig 1**). Apomorphine hydrochloride was dissolved in a solution of 0.9% saline and 0.1% ascorbic acid and was injected sub-cutaneously under the nape of the neck. In each case, the injection volume was 1 ml/kg; solutions of drugs were prepared fresh daily. Out of a total of 8 lesioned rats in the *substantia nigra*, only 3 were selected for the analyses after histological verification.

#### Labyrinthectomy

In brief, a global inner ear lesion was performed with the aid of an operating microscope. A retroauricular approach was used. The entire length of the ventral edge of the meatus was destroyed and the facial nerve sectioned. The tympanic membrane, malleus and incus were removed to expose the pterygopalatine artery caudal to the stapes. This artery was coagulated using an electro-coagulator, the stapes removed and the oval window opened. Finally, the vestibule was destroyed using a suction tube. A post-operative i.m. Terramycin (Pfizer, Paris, France) injection was given (50 mg tetracycline) to prevent infection, and the animals were then left free in normal visual conditions until compensation of the vestibular deficits [12]. Out of a total of 5 rats labyrinthectomized, 3 were tested in this study.

### Locomotor behavior

To follow the movement in three-dimensional space, five digital cameras were used to register sequences of 8 seconds at 200 frames per second. Each of them was placed in specific planes of space so that at least two cameras would be able to capture and follow the same point in movement simultaneously.

**Fig 2:**
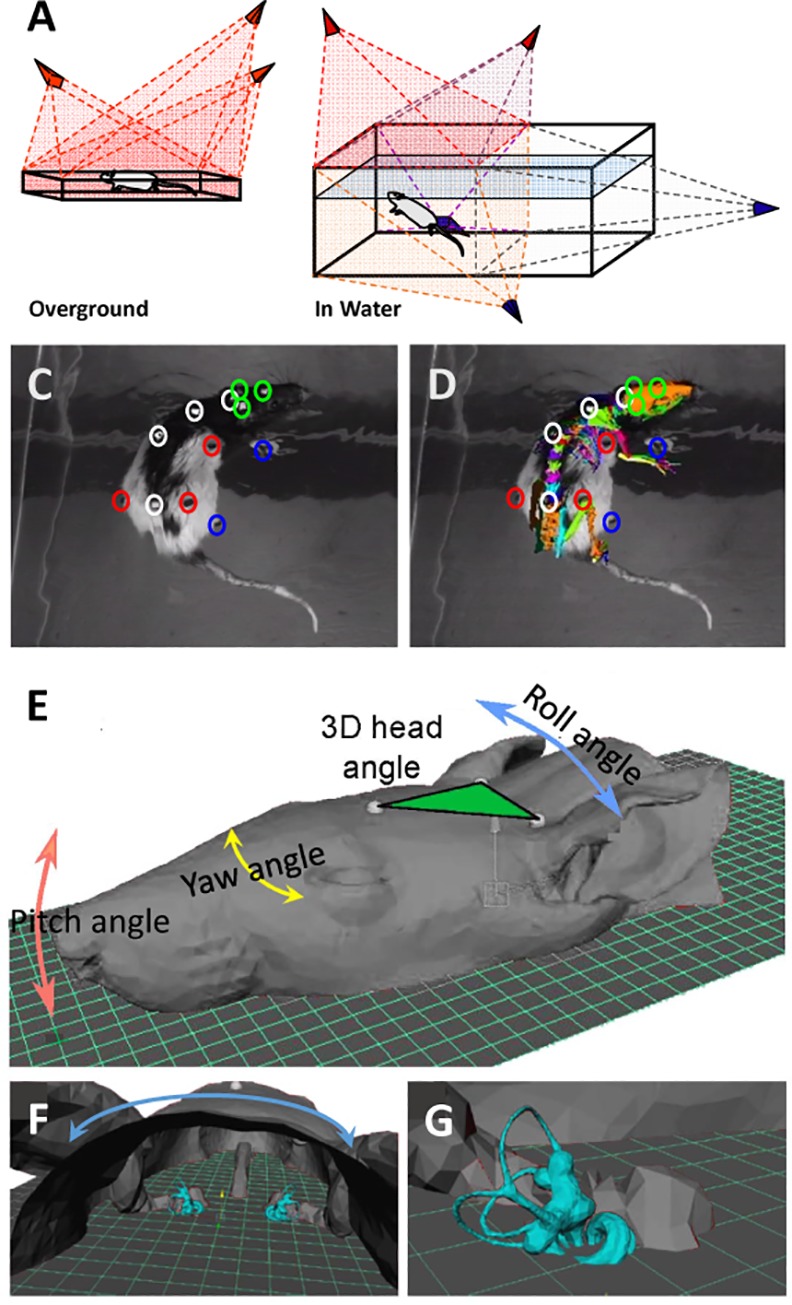
Methods used to measure locomotor behavior and to model the 3D trajectories of the different part of the skeleton. **(A)** Setup used on overground; 3 cameras permit motion capture of the rat on the ground. **(B)** Setup used to capture motion of the rat in the water; 2 cameras capture the movement of the rat over the water, and 3 cameras capture the movement underwater. **(C)** Position of the different markers on the skin of the rat. Green for the head, white for the vertebral column, red for the girdles and blue for the distal part of the limbs. **(D)** The cutaneous markers and their relative positions on the skull. **(E)** Head of the rat and the different angles analyzed (3D angle, Pitch_H_, Yaw_H_ and Roll_H_). **(F)** Occipital view of the head and localization of the three semicircular canals. **(G)** Focus on the vestibular region.

The frequency was selected as a compromise between maximum time resolution and the spatial definition required for a frame-by-frame data analysis. The referenced animal was then first set on a small wood platform 30 x 30 cm, and filmed with three cameras (*Prosilica GE680*) at 200 frames/second (**Fig 2A**). This first step was done to follow the locomotor behavior on the ground. Secondly, each animal was put in a transparent glass pool (80 x 40 x 40 cm) filled with 32 C° water to a height of 25 cm (**Fig 2A**). The rats were delicately positioned at the surface of the water in the center of the tank and filmed at 200 frames/sec with the all five video cameras (3 *Prosilica GE680*, *1 Basler*, *1 SVSi Memview*). Each exercise was done before and after (two minutes after) subcutaneous injection of Apomorphine (APO).

To follow the changes of the skeletal geometry of the individual in the different conditions, fifteen small colored spherical markers (diameter of 5mm) were stuck on the skin of each animal (**Fig 2B and 2C**; **S1 Movie**); 3 on the head (1 between eyes on nasal, 2 close to each ear) to obtain a three- dimensional reference point, 4 on the back (the first between scapulae, and the last one close to the first pelvic vertebrae and 2 evenly spaced between these two markers, to characterize the spine axis), 1 on each shoulder and at the level of each femoro-pelvic junction to complete position of spine, and finally 1 on each foot, to identify the foot movement.

The average velocity of body rotation was determined for each episode and the mean value was then calculated. Quantitative data acquisition was performed off-line using custom software *(Loco 3*.*01)*, and it was used for the 2D tracking of markers in every camera view. Using geometrical camera calibration from an object with known dimension and standard stereoscopic triangulation, the software allows the determination of the 3D Cartesian coordinates of the tracked markers along a sequence. These 3D trajectories were used to calculate various angles describing the skeletal geometry of the animal during locomotor behaviors (circling and pivoting). In addition, 3D anatomical models of the head and skeleton have been aligned with respect to the 3D trajectories of markers. These 3D anatomical models have been collected using an accurate microCT X-ray scanner (50 microns accuracy). This anatomical model permitted identification of the position of the different anatomical markers fixed on the skin of the animal (**Fig 2C**). The 3D anatomical models include a segmentation of the vestibular system, allowing an estimation of the trajectory of the pitch angle of the HSCC (**Fig 2D, 2E and 2F)**. Four WT rats have been scanned. Vestibular systems have been identified, including HSCC. Anatomical locations of head markers have been identified also on the 3D head models. It allowed us to estimate a pitch angle between the plane including the three head markers and the HSCC of 28°±6° (p < 0.01). The head markers allowed us to calculate the pitch, the yaw and the roll of the head. To estimate the pitch angle (Pitch_H_), we calculated the mean angle from the difference of pitch angle between the Marker 1 and 2 and pitch angle of the Marker 1 and 3 (relative to horizontal). The roll angle (Roll_H_) was calculated between Marker 2 and 3 (relative to horizontal), and lastly the yaw angle (Yaw_H_) between segment delimited by Marker 1 and the theoretical midpoint between Marker 2–3 and the segment delimited by this same midpoint marker and the marker stuck between scapulas. Supplemental angles were measured along the spine axis, the yaw angle between the cervical and thoracic column (Yaw_C1_) and the yaw angle between the thoracic and lumbar column (Yaw_C2_). A cumulative value from the two previous yaw gave the yaw of the column (Yaw_C_ = global curvature of the column).The roll angle (tilt) at the shoulder girdle level and the roll angle (tilt) at the pelvic girdle level were also quantified. Statistical comparisons were conducted using one-way ANOVA, and groups were then compared by means of the Bonferroni’s post-hoc test. The vectors, the angles analyzed and the statistics were computed using R [13], *Statistica 6*.*1 (StatSoft*, *Inc*.*)*, and *Prism3*.*02 (GraphPad Software*, *Inc)*.

## Results

### Motor behavior Overground

The control animals (WT) placed on the platform walked along the edges and tried to get out (a theoretical diameter of 38 cm corresponding to the size of the platform was done for reference **Table 1**), then neither description of the locomotion, nor measures on the trajectories of the individual were done in these situations. However the Yaw_H_ and Roll_H_ were estimated from straight ahead locomotion on a horizontal surface (Yaw_H_ = 180°, Roll_H_ = 0°) (**Table 2**), while the Pitch_H_ (28°±6) could be measured from video-radiographies of previous studies on WT rat in movements.

**Table 1:** Characteristics of the trajectory overground and in water for each rat conditions.

		*Diameter of the circling Behavior (cm)*	*Number circle/mn*	*Rotation speed (cm/s)*
**WT**	**G**	38 * ^APO^ (38 for Plateform)	NR	NR
	**W**	76 * ^OH,APO^ (76 for pool)	5 ± 3 * ^APO,UL^	20 ± 11
**6 OHDA**	**G**	28 ± 6	6 ± 2 * ^APO^	9 ± 0.1 * ^APO^
	**W**	28 ± 6 * ^WT,APO^	5 ± 2 * ^APO,UL^	7 ± 1 * ^APO^
**APO**	**G**	24 ±6 * ^WT^	38 ± 6 * ^OH,UL^	47 ± 8 * ^OH,UL^
	**W**	11 ± 2 * ^WT,OH^	31 ± 8 * ^WT,OH,UL^	17 ± 3 * ^OH^
**UL**	**G**	26 ± 12	6 ± 2 * ^APO^	8 ± 2 * ^APO^
	**W**	NR	NR	NR

The mean values (± standard deviation) have been compared for the same support in the other rat conditions (* Acronym in inset represent a significantly difference with other rat condition (one-way Anova with p<0.05, and t-test). WT; Control rats, 6-OHDA; Rats with unilateral degeneration of the whole nigro-neostriatal dopamine neuron system by intracerebral injection of 6-hydroxy-DA, APO; 6-OHDA rats with injection of apomorphine, UL; Rats with a hemilateral labyrintectomy, G; overground, W; in water, SD Standart deviation, NR; non relevant value.

**Table 2:** Change of the different angles (yaw, pitch, roll) measured during locomotion on both supports for each rat conditions.

		YAW _H_ Head vs cervical column (Deg)	PITCH _H_ Head vs cervical column(Deg)	ROLL _H_ Head vs Cervical column (Deg)	YAW _C1_ Cervical vs Thoracic column (Deg)	YAW _C2_ Thoracic vs Lumbar column (Deg)	YAW _C_ Curvature of the column (Deg)	ROLL Shoulder gridle (Deg)	ROLL Pelvic girdle (Deg)
**WT**	**G**	180 * ^OH,APO,UL^ (straight ahead)	28 ± 6 * ^OH,UL^	0 * ^OH,APO,UL^	180 * ^APO,UL^ (straight ahead)	180 * ^OH,APO,UL^ (straight ahead)	0 * ^OH,APO,UL^ (straight ahead)	0	0
	**W**	163 ± 16	12 ± 2	18 ± 2 * ^OH,APO,UL^	155 ± 4	162 ± 12	60 ± 31	4 ± 1	8 ± 2 * ^APO^
**6 OHDA**	**G**	162 ± 5 * ^WT,APO,UL^	14 ± 4 * ^WT,APO^	15 ± 1 * ^WT^	156 ± 2	166 ± 3 * ^WT,APO^	57 ± 1 * ^WT,APO^	0	0
	**W**	159 ± 6	14 ± 5	8 ± 5 * ^WT,UL^	168 ± 3	168 ± 5	46 ± 2 * ^APO^	7 ± 7	5 ± 1 * ^APO^
**APO**	**G**	170 ± 2 * ^WT,OH,UL^	26 ± 3 * ^OH,UL^	20 ± 2 * ^WT^	141 ± 21 * ^WT^	149 ± 8 * ^WT,OH,UL^	80 ± 15 * ^WT,OH^	0	0
	**W**	158 ± 8	12 ± 10	7 ± 6 * ^WT,UL^	151 ± 16	159 ± 3	72 ± 19 * ^OH^	11 ± 11	21 ± 6 * ^WT,OH^
**UL**	**G**	152 ± 6 * ^WT,OH,APO^	12 ± 2 * ^WT,APO^	23 ± 14 * ^WT^	143 ± 40 * ^WT^	166 ± 4 * ^WT,APO^	79 ± 52 * ^WT^	0	0
	**W**	155 ± 17	20 ± 10	360 * ^WT,OH,APO^	123 ± 38	156 ± 16	106 ± 56	NR	NR

Each mean (± standard deviation) angle has been compared with the same angle, same support in the other rat conditions (* Acronym in inset represent a significantly difference with other rat condition (one-way Anova with p<0.05, and t-test). WT; Control rats, 6-OHDA; Rats with unilateral degeneration of the whole nigro- neostriatal dopamine neuron system by intracerebral injection of 6-hydroxy-DA, APO; 6- OHDA rats with injection of apomorphine, UL; Rats with a hemilateral labyrintectomy, G; overground, W; in water, SD Standart deviation, NR; non relevant value.

**Fig 3:**
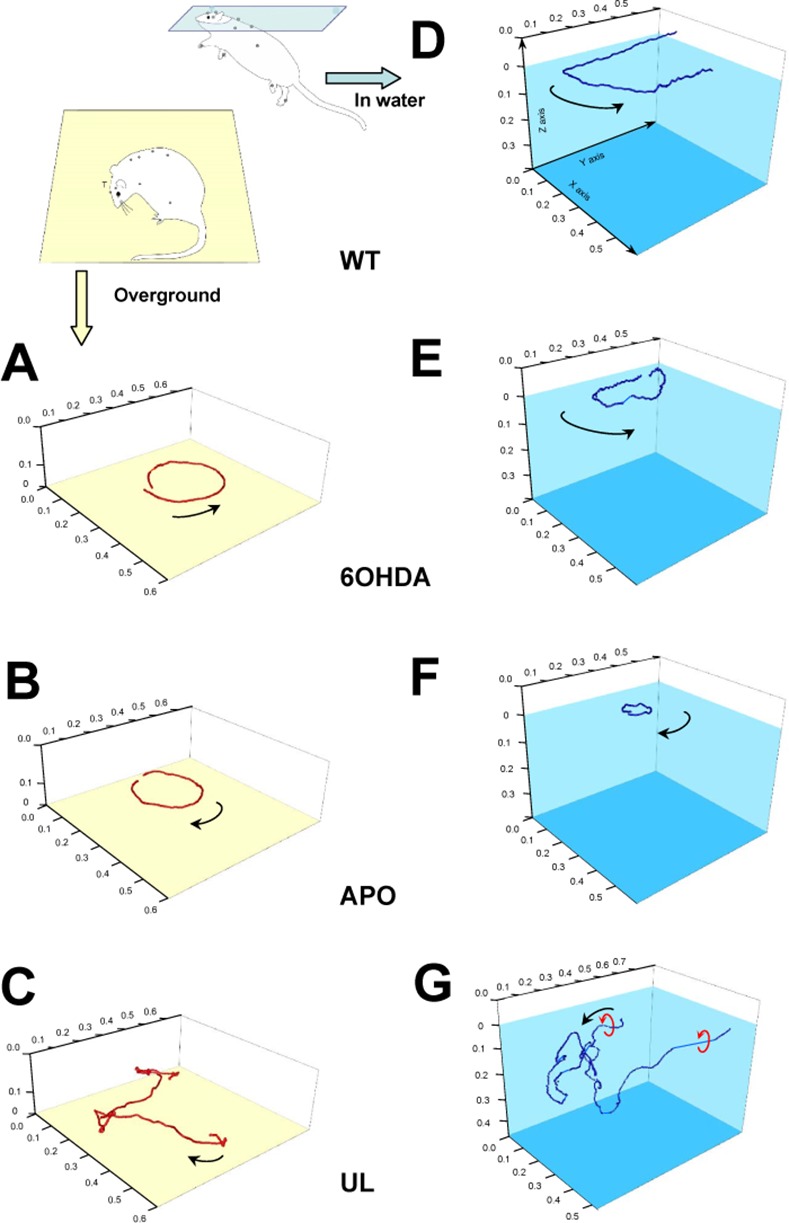
3D head trajectory showing circling and pivoting behaviors overground or in the water. The position and trajectory of the head recorded in the 3 dimensions of space by a multi- camera system during 11 seconds (200 Hz) overground **(A, B, C)** or in the water (**D, E, F, G)**. **(A)** Example trajectory of rat with unilateral degeneration of the whole nigro-neostriatal dopamine (DA) neuron system by intracerebral injection of 6-hydroxy-DA (6-OHDA) overground; The 6-OHDA rats with a lesion on the left side present only anti-clockwise (ipsiversive to the lesion) circling behavior on the ground. The head trajectory is in the horizontal plane, since the control of the head position is supplied by proprioceptive afferents (limbs, vibrisses). **(B)** Example trajectory of APO rats over ground; a systemic injection of apomorphine (APO) to a 6-OHDA rat results in pivoting behavior toward the opposite side of the lesion (controversive to the lesion). This is due to the activation of the supersensitive DA receptors, which are deprived of their DA afferences. As in 6-OHDA, the trajectory of the head is also in the horizontal plane. **(C)** Example of trajectory of left hemi-lateral labyrintectomy rats (UL) over ground; the UL rats explore the environment in a clockwise direction (contraversive to the lesion) with their heads inclined slightly on the lesion side. The trajectory of the head is in horizontal and vertical planes. **(D)** Example of swimming of the control rat (WT). It explored the environment following the edge of the pool. **(E)** Example trajectory of 6-OHDA in the water; the respiratory constraint causes the rat to swim with the snout in a more upward position compared to the ground. The sense of rotation and the diameter of the circle are the same as in over ground. The trajectory of the head is slightly in the horizontal plane **(F)** Example trajectory of APO rats in water. As in over ground, the systemic injection of apomorphine (APO) results in pivoting behavior toward the opposite side of the lesion. The diameter of the trajectory is tighter than 6-OHDA in the water or APO over ground. As in 6-OHDA, the trajectory of the head is in the horizontal plane. **(G)** Example trajectory of hemi-lateral labyrintectomy rats (UL) in water; the anti-clockwise rolling movements (red arrows) were immediately triggered when the individual attempted to swim. The head position varies highly among the three directions of space. Scale units are in meters. Arrows indicate the direction of the movement.

**Fig 4:**
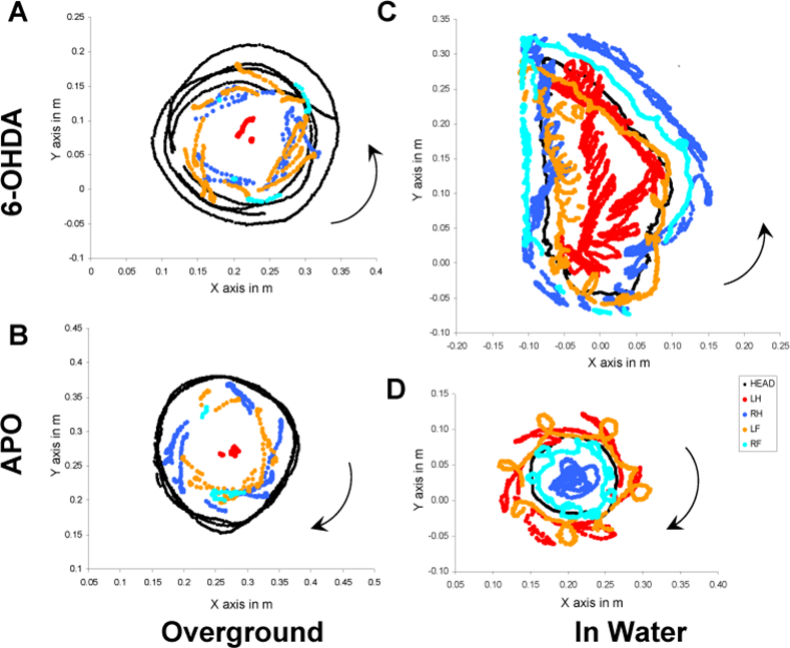
Skeletal geometry in 6-OHDA rat during circling and in APO rat during pivoting over ground and in water. Examples reconstructed from films captured at 200 Hz by a multi-cameras system. **(A, B)** Trajectories of the head and the extremities of the four limbs according to the earth horizontal plane (x,y) during the totality of the sequence of recording (11 s) on the ground. **(A)** The 6-OHDA rat turns in an anticlockwise direction (ipsiversive side to the lesion). The head describes concentric circles and the snout and the vibrissae are in contact with the surface. Limb data are incomplete because landmarks are often hidden by the body of the rat. However, the hindlimb ipsilateral to the lesion is used as a pivot by the rat during its rotation, and the position of the others footfalls are inside the head trajectory. **(B)** All the trajectories of the head of the APO rat (the same shows in A after APO injection) are superimposed, and turn in clockwise direction (contraversive side to the lesion). The hind limb ipsilateral to the lesion is used as a pivot, as in the 6-OHDA, but the other limb shows a backward movement inducing the change of the direction of pivoting. The position of the footfalls of the other limbs do not change, and are also inside the trajectories of the head. **(C, D)** Trajectories of the head and the extremities of the four limbs in the water according to the water surface (x,y) during only one revolution (2.25 s) to facilitate the data visualization. The position of the head in 6-OHDA and APO rat is very horizontal and the snout stays at the limit between the water and the air so that the rat can breathe. **(C)** The 6-OHDA rat turns as overground, in an anticlockwise direction (ipsiversive side to the lesion). The trajectories of the limbs ipsilateral to the lesion are inside or close to the trajectories of the head. The introduction of a lateral component is characterized by trajectories of the limbs contralateral to the lesion outside of the head trajectories. **(D)** The APO rat turns as in over ground in a clockwise direction (contraversive side to the lesion). The forelimbs move out of phase, with a stronger lateral component for the forelimb ipsilateral to the lesion. The hindlimbs present alternate movements, the trajectory of the hindlimb contralateral to the lesion is close to the center of rotation, while the hindlimb ipsilateral to the lesion exhibits the larger lateral trajectory. All of these characteristics induce a faster and tighter rotation of the APO rat in comparison with the 6-OHDA rat. HEAD; marker on the snout. RF; Right forelimb. RH; Right hindlimb. LF; Left forelimb. RH; Right hindlimb. Scale units are in meters. Arrows indicate the direction of the movement. The different axis have the same scale in meter.

Following unilateral destruction of the dopaminergic neurons, the 6-OHDA-rats placed on the platform exhibited a circling behavior ipsiversive to the lesion (**Fig 3A**). The hindlimb ipsilateral to the lesion was never fully lifted off, but several repositionings at the center of the circular trajectory occurred (**Fig 4A**). The others limbs showed alternating movements, with footfalls inside the trajectory of the head. The equivalent diameters of the circles were smaller than the diameter of the platform, and the rotation speed was 9 cm/s (**Table 1**). The position of the head was slightly different of the WT, the head being in a more horizontal position, the Pitch_H_ was less pronounced, the Yaw_H_ was oriented toward the sense of the rotation, as the tilt (Roll_H_) of the head (**Table 2**). The vertebral column was more curved than WT (Yaw_C_ = 57°±1), with a difference of the Yaw_C2_, while the Yaw_C1_ did not change (**Table 2**).

After apomorphine injection, the APO-rats placed on the platform exhibited pivoting behavior contraversive to the lesion (**Fig 3B**). The hindlimb ipsilateral to the lesion was not active, and rotated without having left from the ground, while the hindlimb contralateral to the lesion performed backward steps, and forelimbs acted forwardly and laterally. The footfalls of HLc, FLc, FLi, were as in 6-OHDA inside the trajectory of the head (**Fig 4B**). The diameter of the circle was not significantly smaller than 6-OHDA (but smaller than WT), however the number of circles per minute and the rotation speed were greatly increased 47 cm/s (**Table 2**). The Pitch_H_ of the APO-rats found again the WT value, while the Yaw_H_ orientation was nearby to WT but still different from WT and OH. The tilt of the head was higher than previous in WT and 6-OHDA (**Table 2**). The lateral bending of the vertebral column was higher than WT and 6-OHDA (80°±15), with, as in previous, a significant difference of the Yaw_C2_, while the Yaw_C1_ did not change (**Table 2**).

The unilateral labyrinthectomized rats (UL) as we previously described [1], presented a 3D deficit. Immediately after recovery from general anesthesia, they were lying on the side ipsilateral to lesion and executed rolling movements about the axis of the extended vertebral column. The rotations took place at the junction between the lumbar and thoracic column. About four hours after surgery, thanks to vestibular compensation [14], UL-rats were able to stand up again. When the UL-rats were placed on the platform, they showed a circling behaviour contraversive to the lesion (**Fig 3C**). The diameters of the circles were not different to those of other rats; the rotation speed was slower than APO-rat but not different than the other rats (**Table 1**). However, at rest, they presented a static postural syndrome oriented toward the side of the lesion. This syndrome had two components: a rotation about the axis of the thoracic column caused a lateral tilt of the head-neck ensemble. This head tilt (Roll_H_ = 23° ± 14) was combined with a head rotation about the axis of the cervical column (Yaw_H_ = 152° ± 6); the utricular macula lesion (UT) caused the head tilt, while the horizontal semicircular canal lesion (HSCC) caused its rotation. At last, the curvature of the vertebral column seemed to not be modified in comparison with injured rats, but less stable (highest SD = 52 for Yaw_C_).

In summary, on ground, UL-rats displayed a 3D motor syndrome which concerned their mobility both in a plane related to their basis of support (circling) and to the roll and pitch plane. In contrast, 6-OHDA and APO- rats exhibited a 1D motor syndrome (circling and pivoting) only related to their mobility in a plane related to the support, and the impossibility to move straight-ahead.

### Motor behavior in water

When they were placed in the water, the control rats swam close to the edge of the rectangular pool (**Fig 3D**). The fore limbs were always extended and maintained ahead at the surface, while the hind limbs presented alternate movement as occurs in symmetrical walk. The diameter of the trajectory was compared to the diameter of the pool. The rats, to breathe, maintained their heads above the surface and then modified the Pitch_H_ (12° ± 2) compared to when over ground (**Table 2**). Additionally, as they swam close to the edge, they needed to turn at the corner of the pool, and then modified the different yaw angles (high SD for Yaw_C2-C_).

The 6-OHDA placed in the pool, circled toward the lesion side (**Fig 3E).** The diameters of the circles were smaller than WT, as the rotation speed slower in comparison to WT (**Table 1**). However, the motor behaviour of 6-OHDA rats was not markedly different from the control. When they swam, as was seen in WT, the hind limbs presented alternate movement as occurs in symmetrical walk, while the fore limbs were always extended and maintained ahead at the surface. The hindlimb contralateral to the lesion showed larger lateral movements than the hindlimb ipsilateral to the lesion (**Fig 4C**). It was this difference of movement which caused the circling behaviour in the water. The 6-OHDA, like WT, maintained their head stable above the surface with the same Pitch_H_ and Yaw_H_, while the Roll_H_ was different to the WT (8° ± 5). The curvature of the vertebral column and the Roll plane of the girdles were not different from WT (**Table 2**).

Following apomorphine injection, the sense of rotation reversed (**Fig 3F**) about an axis located at the level of the hind limb contralateral to the lesion. APO-rats swam faster in tighter circles (**Table 1**). Contrary to control and 6-OHDA, they swam using the hind and forelimbs (**S1 Movie**), but the coordination of the limbs did not change and was still alternated. A new lateral component was added in the movement of the limbs, with highest amplitude of the limb ipsilateral to the lesion, which induced the pivoting of the APO rats (**Fig 4D**). The orientation of the head, Yaw_H_, Pitch_H_ and Roll_H_ did not differ from 6-OHDA (Table 2). Accordingly to the tighter circular trajectory, the lateral bending of the vertebral column (Yaw_C_) was higher than in 6-OHDA. The lateral component added by the limb to induce the rotating behavior was concomitant with the change of the roll plane. This movement of the limbs did not change the roll angle of the both girdles, (**Table 1**).

The UL-rats were tested in water three weeks after the lesion, once the circling and the postural deficits observed on ground had recovered. When gently placed in water, UL-rats immediately rolled again about their longitudinal axis toward the side of the lesion and swam away from the surface in random directions (**Fig 3G**), which obliged one to rapidly rescue them. The Yaw, Pitch and Roll angles seem to not be different from the other rats, but the lack of an observed statistical difference could be because these values had the highest variability (**Table 2**).

In water, proprioceptive afferences related to the plane of support were absent, which obliged the rats to rely on vestibular-related information and vision to control their trim. The reappearance of rolling movements and the problems of navigation at distance from the lesion in UL rats, confirmed that proprioceptive information related to the basis of support are instrumental in the compensation of their 3D motor syndromes. They were used as substitutes for the missing utricular afferences, to control posture in the frontal and sagittal planes and also as substitutes for the HSCC afferences to control navigation.

## Discussion

This study, as explained in the introduction, was aimed at choosing between two hypotheses. One hypothesis is that the dopaminergic system exerts three-dimensional control over motricity, as the vestibular system does. Alternatively, the dopaminergic network, together with neurons processing the horizontal canal information, could control the homeostasis of posture and locomotion specifically in one and only one plane of space, *i*.*e*. the plane related to the basis of support. In that case, barrel rolling should never occur, whether at the acute or chronic stage on firm ground or in water. Moreover, circling should have the same characteristics following both types of lesions. Clearly, 6-OHDA and APO-rats never exhibited barrel rolling at the acute stage. They circled at the acute stage of the lesion and continued to do so three weeks later, including in water. In contrast, UL-rats, exhibited both circling and barrel rolling at the acute stage, then only circled on the ground from time to time once compensation had occurred. Furthermore, barrel rolling instantaneously reappeared in water in UL rats, which was not the case in 6-OHDA and APO-rats. That is, the lesion of the dopaminergic system on one side did not compromise trim in the pitch and roll planes, even when proprioceptive information related to the basis of support was lacking as in water. Altogether, these results strongly suggest that dopamine does not exert three-dimensional control of the motor system but regulates postural control in one particular plane of space, the one related to the basis of support. In contrast, as previously shown, the vestibular system exerts three-dimensional control on posture. That is, we show here for the first time a relationship between a given neuromodulator and the spatial organization of motor control.

This result is in good agreement with several previous studies. First, none of the studies investigating navigation in the Morris water maze following depletion of the dopaminergic system by unilateral or systemic injection of 6-OHDA or MPTP have mentioned long lasting difficulty in swimming and even less turning. That is, trim in the frontal and sagittal plane is preserved [15–22]. In contrast, mutant rats and mice with abnormal utricular function are known to be unable to swim [23–25] due to barrel rolling. Second, Ishiguro and collaborators [26] studied the Bronx Waltzer (bv) mouse displaying major hearing and vestibular dysfunction and showing a remarkable repetitive circling behavior. They investigated whether this behavior was caused by the asymmetry of striatal function by observing the behavior of the bv mice following microinjection of dopamine D1 agonist, into the striatum ipsilaterally and contralaterally to the preferred direction of rotation separately. We retrieved the same result in Isk-/- mice, years later in a study focused on vestibular neurons in this mutant [27]. A recent paper by Armstrong and collaborators (2015) [28] showed that caspase deficient mice with impaired horizontal semicircular canal function and intact otolithic function circle but do not exhibit barrel rolling. Altogether, our results and those of previous studies show that a congenital lack of vestibular function results in a major asymmetry in striatal function, which in turn is causally related to circling.

Finally, this paper confirms unpublished information on the behavior of UL vertebrates and published ones on the behavior of vestibular mutants [23–25], when placed in a tank of water. After a recovery time from the lesion, UL rats displayed erratic navigation, failed to find the surface and exhibited rolling movements, which confirmed that, on ground, proprioceptive information related to the basis of support substituted for the missing vestibular afferences. It explains a- the disappearance of the rolling episodes in UL rats on ground following vestibular compensation; b- the resemblance of their behavior with 6-OHDA and MPTP rats, as only episodic circling episodes persisted.

Importantly, the pivoting behavior of the 6-OHDA and APO rats in water was markedly different from that observed on ground: the whole body rotated in bloc in the water instead of pivoting about the axis of the outer hind limb as occurs while on ground and the pattern of the limb movements was quite different during swimming and walking. It shows that the dopaminergic system does not modulate motor control at an immediate pre-motor level. It more likely participates, together with the information encoded by the HSCC, in the alignment of the posture of a multi-articulated skeletal system at rest and in the perception of the straight-ahead direction during navigation.

The selective modulation of postural control by dopamine in a plane related to the basis of support is of clear interest at the clinical level. For instance, one of the first signs of Parkinson’s disease is the inability of patients to turn their body about its longitudinal axis when lying in bed. Similarly, at a more advanced stage of the disease, patients get stuck in front of an obstacle. The inability to rotate in a plane related to the basis of support could be at stake in both cases. Another point to examine is the nature of the neuromodulation in the frontal and sagittal planes. Turning has been described for 30 years following intraventricular perfusion of various peptides such as opioids and arginine vasopressine, which could be an indication. Finally, if different types of neuromodulation occur in the different planes of space, the implication in case of pathological mismatch between these different types of modulations remains to be explored.

## Supporting Information

S1 Movie Pivoting behaviour and 3D head trajectory in the water. Dorsal / lateral and external / internal views of an APO rat pivoting in water. The cutaneous markers allowed following the trajectories of different anatomical units during the swim. Here the trajectory of the snout (in black), and the extremity of the left fore limb, shows that the head is maintained in a horizontal plane and describes a circular trajectory, and the fore limb moves laterally, with alternate movement. 3D anatomical models of head and skeleton have been aligned with respect to the 3D trajectories of markers. These 3D anatomical models have been collected using an accurate micro-CT X-Ray scanner (50 microns accuracy). The 3D anatomical models include a segmentation of the vestibular system, allowing an estimation of the trajectory of the pitch angle of the HSCC.
(AVI)
